# Implementation process and outcomes of a mental health programme integrated in primary care clinics in rural Mexico: a mixed-methods study

**DOI:** 10.1186/s13033-020-00346-x

**Published:** 2020-03-16

**Authors:** Georgina Miguel-Esponda, Nathaniel Bohm-Levine, Fátima Gabriela Rodríguez-Cuevas, Alex Cohen, Ritsuko Kakuma

**Affiliations:** 1 Centre for Global Mental Health, Department of Population Health, Faculty of Epidemiology & Population Health, London School of Hygiene & Tropical Medicine, Keppel Street, London, WC1E 7HT UK; 2 University of California, San Francisco, USA; 3 Compañeros en Salud, Jaltenango de la Paz, Chiapas Mexico

**Keywords:** Depression, Anxiety, Mental health programmes, Implementation, Process, Implementation outcomes, Rural, Primary health care, Mexico, Mixed-methods

## Abstract

**Background:**

Policies and programmes in Mexico promote the integration of mental health services into primary health care (PHC), however these services remain largely unavailable in the country. Since 2014 a non-governmental organisation has delivered a mental health programme at PHC clinics in the state of Chiapas, in partnership with the local Ministry of Health (MoH). The programme provides mental health services based on the mhGAP guidelines through multiple implementation strategies, including programme financing, infrastructure strengthening, high-intensity training, and supervision. This study aimed to examine the implementation process and outcomes of this mental health programme to understand the extent to which mental health care integration has been achieved and to identify the successes and remaining challenges in order to inform the development and implementation of similar programmes.

**Methods:**

We used a mixed-methods convergent design. Quantitative data for the period between December 2016 and December 2017 were extracted from the organisation’s health information system to capture process indicators, including the amount (dose) and quality (fidelity) of services delivered. We conducted two focus groups and 24 semi-structured interviews with health providers and managers to ascertain implementation outcome data: penetration, fidelity, acceptability, appropriateness and feasibility. Quantitative and qualitative data were analysed using descriptive and framework analyses, respectively.

**Results:**

During the study period, health providers delivered mental health consultations to 486 adults diagnosed with a mood or anxiety disorder. Programme fidelity was limited given that talk-based interventions, which are required in all consultations according to programme guidelines, were only provided in 24% of consultations. Only 42% of service users attended more than 50% of scheduled mental health follow-up consultations, which also hindered fidelity. Low attendance is partially attributed to limited programme appropriateness, given that interventions to address social risk factors are not available. High levels of acceptability and feasibility enabled through strong support from the organisation were key programme strengths.

**Conclusions:**

Mental health programmes at PHC can be implemented when adequate support and supervision structures are in place, and key resources are available. There is an urgent need for health systems strengthening to support efforts to provide mental health care, and to link PHC with locally-relevant social interventions.

## Background

Since the late 1990s, Mexico has been working to shift from a heavily centralised and institutionalised mental health system to a community-based model, in order to increase access to quality services and to protect the human rights of people with mental disorders [1–3]. The Mexican mental health policy promotes three main elements: (1) integrating mental health services in general health services, (2) increasing human resources, budgets and quality of mental health care, and (3) increasing health promotion and advocacy activities [4]. More recently, Mexico’s Action Program in Mental Health (2013–2018) specifically aimed to improve the coverage and quality of mental health services through the integration of mental health care into primary health care (PHC) [2]. Despite having progressive policies and programmes supporting the integration of mental health into PHC, in Mexico mental health care is still mainly delivered at psychiatric institutions, and it is only available in 30% of PHC clinics in the country [3]. A national epidemiological study found that 20% of people diagnosed with a mood disorder and 10% of people diagnosed with an anxiety disorder accessed care, and only 50% of people who accessed specialist services received minimally adequate care [5]. Resource constraints are important barriers to the improvement of mental health care in Mexico, where only 2% of the health budget is allocated to mental health [3], there are 0.67 psychiatrists per 100,000 people [6], and the few services are hampered by staff and medication shortages [4].

Better understanding of the challenges surrounding translation of policy into practice is crucial to the improvement of mental health care. This study examines the implementation process and outcomes of a relatively young PHC mental health programme in Chiapas, a low-resource, rural state in southern Mexico. Since 2011, Compañeros En Salud (CES), a non-governmental organisation (NGO) and sister organisation of Partners in Health, has been supporting 10 PHC clinics in rural Chiapas in a partnership with the local Ministry of Health to improve the delivery of general health services [7]. In 2014, the mental health programme was introduced in these clinics. Prior to this, mental health services were only available more than 6 h away in the state capital [4, 7].

Our study assesses the implementation of the CES programme in order to understand the extent to which it has achieved the integration of mental health into PHC as outlined in Mexico’s mental health policies, and to then explore the strengths and limitations that determine the success or failure of integration in this context. Specific research questions include:

1. To what extent are mental health services from the CES programme delivered as intended?
2. What are the perspectives of programme managers and providers regarding its penetration, fidelity, acceptability, appropriateness, and feasibility?
3. What are the key strengths and remaining challenges to the implementation of the CES mental health programme?

## Methods

### Setting

Of the approximately 5 million inhabitants of Chiapas, 50% per cent live in rural areas [8] and 77% in poverty [9]. Depressive and anxiety disorders are among the top 10 causes of disability in the state [10]. Mental health services are mainly accessed through either the psychiatric hospital or an ambulatory clinic located in the state capital [4]. The 10 PHC clinics supported by CES are each staffed by one medical doctor (MD) and, when possible, one nurse. Each PHC is located in one of the 10 communities of the mountainous Sierra region, approximately 6–8 h from the state capital. Each community has ~ 1000 inhabitants, most of whom live in extreme poverty [11].

### CES mental health programme

CES aims to strengthen the PHC system to improve access to quality health care. The organisation facilitates the delivery of general health services (including mental health) in 10 PHC clinics through the following implementation strategies: (1) programme financing, (2) capacity building of medical doctors (MDs) through high-intensity training and on-site supervision, (3) printed materials for clinical decision-making, (4) monitoring through a health information system (HIS), (5) ensuring medication supply, (6) “community-based accompaniment” [12] by community health workers (CHWs) and (7) support for referrals to specialist services. Previous studies have found these strategies to be effective for the provision of care for various health conditions in other low resource settings [13].

**Fig. 1:**
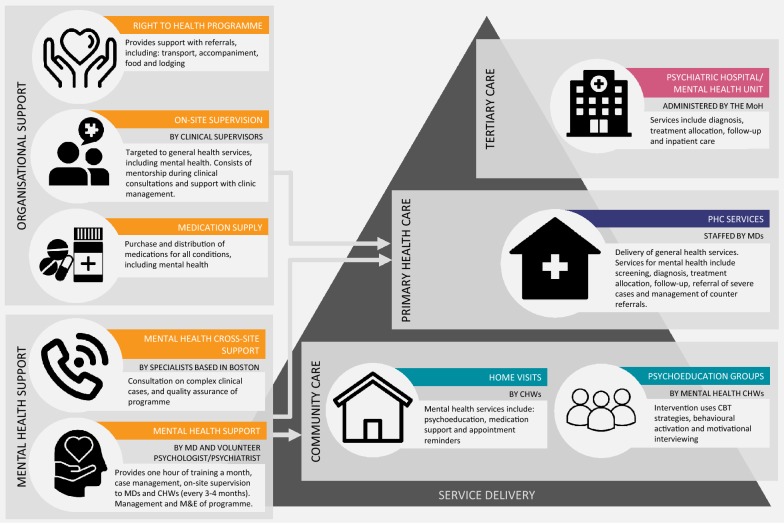
CES mental health programme: organisational support and service delivery

For mental health, a coordinator oversees the delivery of mental health services and capacity building activities, and provides support for the management of complex cases. All mental health services are delivered by MDs, who rotate every year, in the clinics, and CHWs in the community. MDs are contracted by the MoH but appointed by CES. CES also provides an additional stipend to MDs. Services are designed according to mhGAP (Version 2.0) [14] adapted clinical guidelines and include case identification, diagnosis, pharmacological treatments (i.e. prescription and supply of antidepressants, benzodiazepines, mood stabilisers and antipsychotics), individual and group talk-based interventions, and home visits. A full description of the programme can be found in Fig. 1.

### Study design

We employed a mixed-methods convergent study design. Between May 2017 and February 2018, we collected quantitative and qualitative data simultaneously and compared the complimentary data sets to more holistically understand the CES mental health programme implementation. To integrate the quantitative and qualitative data, we identified common findings across the data sets and compared them to determine how these confirmed, disconfirmed or enhanced understanding of each other [15].

### Quantitative data

#### Sample

We included all service users registered in the HIS (i.e. electronic medical records stored in CES’ Microsoft Access database) who received a consultation at any of the 10 CES supported clinics and met the following criteria: (1) 18 years of age or older, (2) diagnosed with a mood (i.e. depression, dysthymia or bipolar disorder), anxiety or mixed disorder, and (3) had attended the clinic at least once between December 2016 and December 2017 to receive services for a mental health complaint. We included people diagnosed with a mood or anxiety disorder as the CES programme specifically targeted this group, and 95% of the programme’s service users received treatments for these disorders [7]. We excluded children, adolescents and those who were diagnosed with psychosis or had experienced psychotic symptoms given that these service user groups require significantly different services.

#### Quantitative data collection

We extracted de-identified routinely collected service user data from the organisation’s HIS for the period between December 2016 and December 2017. Extracted data included sociodemographic indicators (sex, age, and community of residence), clinical characteristics (diagnosis, PHQ-9 and GAD-7 scores, presence of comorbid conditions, treatment allocated, medication prescriptions, and months in treatment) and clinical notes (MDs records at diagnosis and follow-up consultations). Less than 1% of data was missing. We selected dose and fidelity as relevant process indicators based on the Medical Research Council (MRC) guidance on the evaluation of complex interventions [16] and developed indicators based on the programme’s guidelines (Table 1).

**Table 1:** CES programme guidelines for mental health service delivery and process indicators

Mental health service	Programme guidelines for the treatment of mood or anxiety disorders	Indicator and description	Data source for indicator
Diagnosis	Performed using (1) the score of the 9-item Patient Health Questionnaire (PHQ-9) [17] for mood disorders or the 7-item Generalized Anxiety Disorder scale (GAD-7) [18] for anxiety disorders and (2) a clinical assessment (i.e. exploration of key symptoms, medical history, and relevant life events)	Fidelity to diagnosis guidelines: proportion of service users diagnosed according to guidelines	Coded from content in clinical notes
Treatment allocation	Pharmacological treatment is allocated when PHQ-9 [17] or GAD-7 [18] score is 15 or above unless user rejects medication or clinician decides to reassess need in a second appointment; if 14 or less, medication is not prescribed until reassessment at second appointment; psychoeducation or other talk-based intervention is provided in all cases	Fidelity to treatment allocation guidelines: proportion of service users diagnosed according to guidelines	Coded from content in clinical notes
Follow-up	Follow-up provided until remission (at least 6 months with no symptoms)	Dose of mental health follow-up consultations: proportion of service users who attended more than 50% of programmed monthly consultations	Dates recorded on clinical notes
Clinical assessment at follow-up	Clinical assessment at follow-up done through: (1) the use of PHQ-9 [17] or GAD-7 [18], (2) key symptom exploration, and (3) life event exploration	Fidelity to clinical assessment guidelines at follow-up: proportion of service users who receive a clinical assessment according to guidelines	Coded from content in clinical notes
Treatment allocation at follow-up	Proportion of service users who receive counsel or advice, or a talk-based intervention at follow-up	Fidelity to treatment allocation guidelines at follow-up: Proportion of service users who receive counsel or advice, or a talk-based intervention at follow-up	Coded from content in clinical notes

#### Quantitative data analysis

We used descriptive statistics to summarise the sociodemographic and clinical characteristics of the clinical sample. The clinical notes were coded using a pre-established system developed using programme guidelines. The coding system can be found in Appendix 1. One researcher coded all clinical notes and a second researcher independently coded a random sample of 20% of these notes to check the reliability of the coding. The coding was in agreement in 87.4% of cases. We then calculated means and proportions to describe process indicators. All analyses were conducted in RStudio (Version 1.1.453).

### Qualitative data

#### Sample

For the qualitative data collection, we used a convenience sample. During the study period there were a total of 14 MDs and 13 nurses working in CES supported clinics, and 10 clinical supervisors, two programme coordinators, six administration staff and five organisation directors working for CES. We aimed to include all members of staff working for CES and CES supported clinics as they were all directly or indirectly involved in the programme implementation, however we were only able to include those available for a face-to-face interview. The sample was comprised of 12 MDs who delivered mental health services, eight nurses who were not directly involved in the mental health programme but supported the delivery of general health services, four clinical supervisors who provided monthly on-site support to MDs, the mental health programme coordinator, the maternal health programme coordinator who oversees the mental health care of women during or after pregnancy and two organisation directors who oversee the overall activities of CES.

#### Qualitative data collection

We collected qualitative data to assess the implementation outcomes (penetration, fidelity, acceptability, appropriateness, and feasibility) selected according to the framework developed by Proctor and colleagues [19]. Two experienced Spanish-speaking qualitative researchers conducted two focus groups (with two directors, two programme coordinators, and six MDs), and 24 semi-structured interviews (with 12 MDs, eight nurses, and four clinical supervisors). Guides for data collection can be found in Additional file 1: Appendix S2.

Data collection took place in the main office of the organisation, clinics or residences of participants, according to their preference and depending on the availability of a private space. All interviews and focus groups were audio recorded, except in two instances when consent was not provided, so detailed notes were taken. All audio recordings were transcribed verbatim by bilingual researchers. GME checked the quality and accuracy of these transcriptions.

#### Qualitative data analysis

Framework analysis was utilised to analyse the qualitative data. We followed a process of (1) data familiarisation, (2) coding, (3) development of an analytical framework, (4) framework application and (5) interpretation [20]. We used pre-established definitions of implementation outcomes [19] to develop the analytical framework. The analysis was conducted in Spanish. Two bilingual researchers familiar with the context translated relevant quotes to English. The accuracy of these translations was assessed by a group of independent bilingual researchers, and changes were made if needed.

## Results

### Penetration

We used quantitative and qualitative data to explore the extent of penetration of the CES mental health programme, defined as the extent to which the programme activities have been integrated into the organisation and the PHC clinics. According to clinical supervisors and MDs, training and supervision for mental health care are delivered as part of a general curriculum that aims to support MDs in all areas relevant to PHC, including maternal health, nutrition, chronic conditions and infectious diseases. All participating MDs reported providing mental health services at PHC clinics, which include diagnosing, prescribing pharmacological treatment and providing talk-based interventions.

Between December 2016 and December 2017, MDs delivered at least one mental health consultation to 486 adults diagnosed with a mood or anxiety disorder (Table 2). The majority were women (84.4%), around a third were between 18 and 29 years old (34.5%), and two-thirds were living less than 30 min away from the clinics (66.3%). Most were diagnosed with a mood disorder (68.2%), about half were experiencing severe symptoms at diagnosis (50.9%) and 16.7% had a comorbid physical condition (i.e. diabetes, cardiovascular diseases, epilepsy, pregnancy, or asthma). Almost half of service users only received pharmacological treatment (44.6%). The majority did not have a community health worker allocated (82.9%) and had been receiving services for more than 6 months (> 70%).

**Table 2:** General characteristics of the clinical sample (n = 486)

	Total	
	N	%
Sex		
Female	410	84.4
Male	76	15.6
Age		
18–29	166	34.5
30–39	137	28.5
40–49	74	15.4
50–59	57	11.9
> 60	47	9.8
Residing 30 min or less from clinic		
No	164	33.7
Yes	322	66.3
Diagnosis		
Mood disorders	331	68.2
Anxiety disorders	127	26.2
Mixed	27	5.5
Severity at diagnosis (according to PHQ-9 or GAD-7)		
Severe	214	50.9
Moderate	121	28.8
Mild	60	14.3
Minimal	25	5.9
Other medical conditions		
No	398	83.3
Yes	80	16.7
Type of treatment		
Both	129	32.7
Pharmacological	176	44.6
Talk-based interventions	90	22.8
Community health worker assigned		
No	403	82.9
Yes	83	17.1
Months in treatment		
1–6	139	28.6
7–12	191	39.3
13–24	75	15.4
25–36	44	9.0
37–50	37	7.6

Most participants highlighted that the support offered by CES for the mental health programme through the appointment of a programme coordinator and funds for the purchase of medications has been key to its penetration. This support acknowledges the importance of addressing the mental health needs of service users at PHC services, provides necessary resources, and builds capacity to do so:

“*The organisation facilitates things because if it was not for its initiative to treat mental health, there would not be any services for mental health. If I were only supported by the Ministry of Health, I would not know what to do with mental health* [service users].” (Com6X, MD, male)

### Fidelity

We used both process indicators and qualitative data to assess fidelity, i.e. the extent to which the programme was delivered as intended. In this section, we explore fidelity to guidelines, dose of services delivered, and quality of services.

### Fidelity to guidelines

**Fig. 2:**
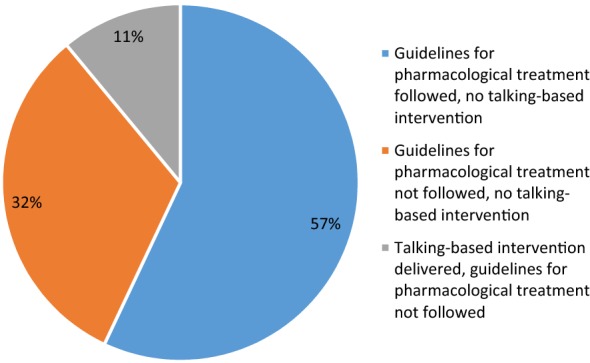
Reasons for lack of treatment fidelity (N = 350)

MDs at CES supported PHC clinics identified service users with potential mood or anxiety disorders, and made diagnoses. 63% of service users were diagnosed according to programme guidelines, 25% did not undergo a clinical assessment, 5% did not complete an assessment scale, and 7% had missing data that prevented determination of the diagnostic process. MDs also provided both pharmacological and talk-based interventions at the clinics. Treatment was allocated in full accordance with guidelines for 28% of service users. Of the 72% (N = 350) service users that were not delivered treatment according to guidelines, in the majority of cases this was due to a lack of talk-based interventions (Fig. 2).

Of the 1770 mental health follow-up consultations delivered, MDs used a scale to assess symptoms in 76% of consultations, and further explored key symptoms and relevant life events in 52% and 41% of consultations respectively. A talk-based intervention was provided in 24% of mental health follow-up consultations.

The majority of MDs and clinical supervisors report finding the materials available for the delivery of mental health services, such as the guidelines and other aids to provide talk-based interventions, as useful. Materials offered helpful reminders and made MDs feel more comfortable providing these treatments. However, about a third of MDs reported that guidelines remained under review by the clinical director for many months and, in a few instances, they were not available in a printed format, both of which made it difficult to access relevant information in a timely manner, negatively impacting fidelity.

#### Dose of services: proportion of attendance to follow-up consultations

**Fig. 3:**
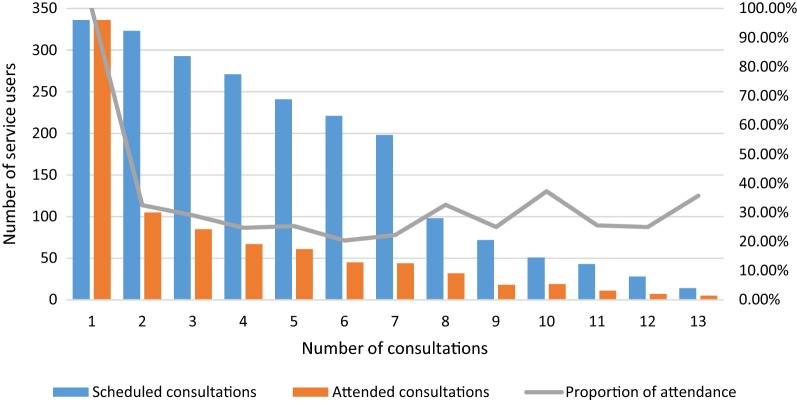
Number of mental health consultations scheduled and attended per service user (N = 336)

Less than half (41.6%, n = 202) of service users attended more than 50% of their corresponding follow-up consultations. To calculate the rate of non-attendance, we analysed data for 335 service users that were enrolled in the programme during the study period. All service users attended their first consultation, but only 20%–37% of subsequent consultations were attended (Fig. 3).

Attendance at follow-up consultations was an important challenge to programme fidelity. A few providers perceived that low attendance is an indicator that some service users are not benefitting thoroughly from the mental health services:

“*I’m not very convinced that* [the treatment impact] *will be a long*-*term thing. Because many times there is low adherence.* [Service users] *come once, and perhaps they let out everything they have been carrying since who knows when. They feel better* […] *and then they don’t come back. I’ve seen it with some patients, it’s a cycle of maybe two, three months and* [then] *they come back because* [the cycle starts] *again.”* (Com1X, MD, female)

Low attendance could be attributed to the limited appropriateness of the programme to the service users’ needs. The lack of social services, difficulties in the communication between providers and users, and distance to clinics were some challenges identified by health providers (see Appropriateness section).

#### Quality of services

The qualitative data also provided insight into barriers to the delivery of quality services. Most MDs reported difficulties providing mental health services at the beginning of their placement, since they received virtually no mental health training in medical school. MDs mainly expressed concerns regarding the delivery of talk-based interventions, since these require skills for which they have not been trained. Most MDs worry that the talk-based interventions that they are providing are of poor quality and hence not useful to service users. However, experience along with exposure to monthly training and supervision were reported to help improve the quality of services:

“*I can’t say that there’s something in particular that has made* [me] *improve. I think that it’s been a bit of everything. The courses. When* [the programme coordinator] *was here and sent me some articles* […] *and the experience in general.*” (Com7X, MD, male)

### Acceptability

We used qualitative data to explore the level of acceptability, i.e. the degree of agreement or satisfaction, of health workers with the CES mental health programme. This was discussed in relation to involvement in training and supervision, delivery of mental health services, and management of the needs of the service users.

All MDs acknowledged the need for mental health care and their limited knowledge and skills to provide it, therefore they were open to receiving training to deliver it. Acceptability from health professionals came from a sense of responsibility to provide needed pharmacological and non-pharmacological treatments. MDs recognised that they were the only personnel available to provide mental health care due to human resource shortages, and the difficulties in accessing other mental health services:

“*I know that probably what they are going to tell me, or what they come to express, they cannot tell to anyone else. If I do not listen to them, no one else will.* […] *I think it is that commitment.”* (Com1X, MD, female)

However, according to about half of the MDs, the needs of mental health service users were perceived as challenging. Service users have problems that MDs are not used to treating and require lengthy talk-based support which can be difficult to offer due to time constraints and the emotional effort they entail. MDs feel they are treating people going through a large amount of social suffering, whose health is affected by social factors which they cannot address. Limited skills from MDs to deal with these challenges led to a sense of low self-efficacy, which affected acceptability:

“*I asked the questions, but I felt my patients did not find anything that I was doing helpful. I think they felt the same.* […]*My first mental health consultations were chaotic and disorganised. They impacted me because I felt useless and powerless in the face of [service users’] extraordinary problems.”* (Com10X2, MD, male)

MDs received positive validation of their work when they could observe positive outcomes in service users, which also improved acceptability:

“*There are days when you are tired, but when you see a patient is improving or that they are better able to do things in their daily lives* […] *that gives you the energy you need.”* (Com7X, MD, male)

### Appropriateness

We explored perceived appropriateness from the perspective of the health workers (i.e. the fit, relevance or compatibility, of the mental health programme to their needs and those of service users) through qualitative data. Several topics were discussed: the extent to which mental health guidelines and materials fit the needs of MDs in the clinic and during the consultation, the fit between capacity building needs of MDs and actual training and supervision available, and the appropriateness of the programme for the needs of service users. Regarding mental health guidelines and materials, all MDs reported that these are easy to use within their daily practice as they are presented in a concise and simple manner, and also use language that is easy to understand for both clinicians and service users.

In terms of capacity building, all MDs reported that training sessions were helpful but insufficient to develop the skills that mental health consultations require. Supervision and training delivered on site by specialists, although limited due to human resource shortages, were seen as more appropriate for MDs’ needs as they allowed MDs to observe what tools and techniques are used to approach real-life scenarios and further apply them in their practice. Finally, according to all participants, many of the service users have mental health needs that arise from social circumstances, such as economic insecurity and exposure to gender-based violence or trauma, which cannot currently be addressed due to the lack of social services and targeted treatments:

“*One of my patients suffered from sexual violence* […] *If we were in, say, Denmark,* […] *my role would be different. I would be a health provider who would do the first contact and behind me would be a large and prepared team with a lot of resources to give my patient better care than I can do on my own, not because I do not want to give her better care but because I do not have the tools to do so.”* (Com10X2, MD, male)

Even though scales like the PHQ-9 have been validated for this population and talk-based interventions have been developed to respond to users’ needs, using these tools during the consultations can be difficult due to differences in culture and language mannerisms between providers and service users. A clinician explained the following about the difficulties of using the PHQ-9:

“*Sometimes the definition of sadness is relative and in each consultation you have to remind people what each thing means.* […] *It is possible that it is a communication issue.”* (Com7X, MD, male)

In settings with limited infrastructure and high levels of poverty, community-based and outreach services may be more appropriate compared to services in PHC clinics, as many service users face numerous challenges, such as long and costly journeys, to access services at the clinics:

“*For example, if patients come from* [the community] *they have to pay* [for] *a trip. The distance* [is a difficulty] *as well because it is very far and they have to walk. Some patients have told me that they were not able to find a car, and they had to walk up the hill, for* […] *many hours, like 3 or more.”* (Com1W1, nurse, female)

### Feasibility

We used qualitative data to explore health workers’ perspectives related to the extent to which the programme was feasible, i.e. could be implemented within this particular health setting and context. The support and resources available from CES, and time and specialist human resource shortages were discussed.

Delivering mental health services at included PHC clinics was deemed feasible to a certain extent. Providers reported the support structure provided by CES makes the delivery of services possible. In this sense, all MDs and clinical supervisors highlighted that the CES mental health coordinator manages training and supervision, provides advice when dealing with difficult cases, and helps coordinate referrals to other services in the state. In terms of resources, important and complementary aspects are the availability of printed materials to provide mental health treatments and pharmacological treatments, which are provided by CES:

“*In certain cases, you need medication and if we do not have* [any] *its worrying because* […] *the closest pharmacy is hours away. Or you can have the best medications available but if you are not trained to know how to use it, then it is useless.”* (Com10X, MD, female)

Most MDs emphasized the importance of supervision in enabling them to work in the PHC clinics. Supervisors help them with clinical decision-making but also help them deal with the frustration caused by large workloads, the lack of efficient referral systems, and, on a personal level, living in a remote community, far from relatives and friends, and with limited capacity to communicate with them:

“[…]*They come and help me in my work every month. I think without the supervision I would not be able to solve many problems* […]*. It helps a lot that* [my supervisor] *comes and listens to me, personal problems with my friends, my family, everything, about here, the community, how I feel. Both personally and professionally, the supervision is helpful*.*”* (Com5X2, MD, male)

However, the limited knowledge and experience of clinical supervisors in treating mental health conditions was considered a barrier. Since clinical supervisors had no specialist training in psychiatry or psychology, a few MDs felt that they were no better equipped than them in providing mental health services. Moreover, most clinical supervisors identified their lack of training to mentor others in the development of skills relevant to the provision of mental health services as a challenge:

“*I think we need a monthly or bimonthly class to learn what we can do to improve our supervision of mental health […] so that we are told what the MDs are doing well and what they can improve.”* (Sup4, clinical supervisor, male)

We identified two key challenges to the delivery of services within the programme: (1) time constraints coupled with the many competing priorities present at the clinics, and (2) the limited availability of specialists to provide mentorship to MDs. A common concern amongst supervisors was the difficulty of providing good quality support in all areas due to the time constraints and the numerous requirements of each health programme managed at the PHC clinics:

“*It is very difficult to deliver quality […], so I think that something that happens is that each person delivers quality and focuses on what they care about the most or on what they feel the most competent in or on what they feel can help the* [MD] *the most because you cannot give quality in everything, and it is obvious because there are too many tasks.”* (Sup2, clinical supervisor, male)

MDs also report they have to allocate time and effort to complete many different activities. The majority of MDs report frustration with not being able to allocate more time to look for service users who have not returned to the clinic or to conduct home visits, and also consider it unfeasible to complete all the tasks and paperwork that are required by CES and the Ministry of Health. The time available for each consultation is an issue according to most MDs as in many cases service users require services for more than one health complaint:

“*I think the majority of mental health patients should have longer consultation times because you have to do a lot with them,* […] *apply the PHQ*-*9, check that there are no adverse effects from the medication,* […] *check for physical things, but the most important is that it is the time the patient has to talk, unload, and also the time that psychoeducation requires.* […] *I cannot give that in 15 to 20* *min.”* (Com3X, MD, female)

All MDs and clinical supervisors perceive there is a need for more involvement of either psychologists or psychiatrists to improve the training and supervision and also to advise on difficult cases. Related to this, they report that due to their limited experience in delivering treatment for people with mental disorders and the lack of secondary and tertiary services available for service user referrals, people who have complex symptomatology and require psychotherapy are left with inappropriate care:

“*We are lacking trained professionals like psychologists or psychiatrists that can give us feedback and advice.* […] *And the fact that there are no mental health specialists in the state to refer to or to get support from also makes things very difficult because we have seen that what makes our work easier is to have a support structure and we do not have it at other levels of care.”* (Sup1, clinical supervisor, female)

## Discussion

**Fig. 4:**
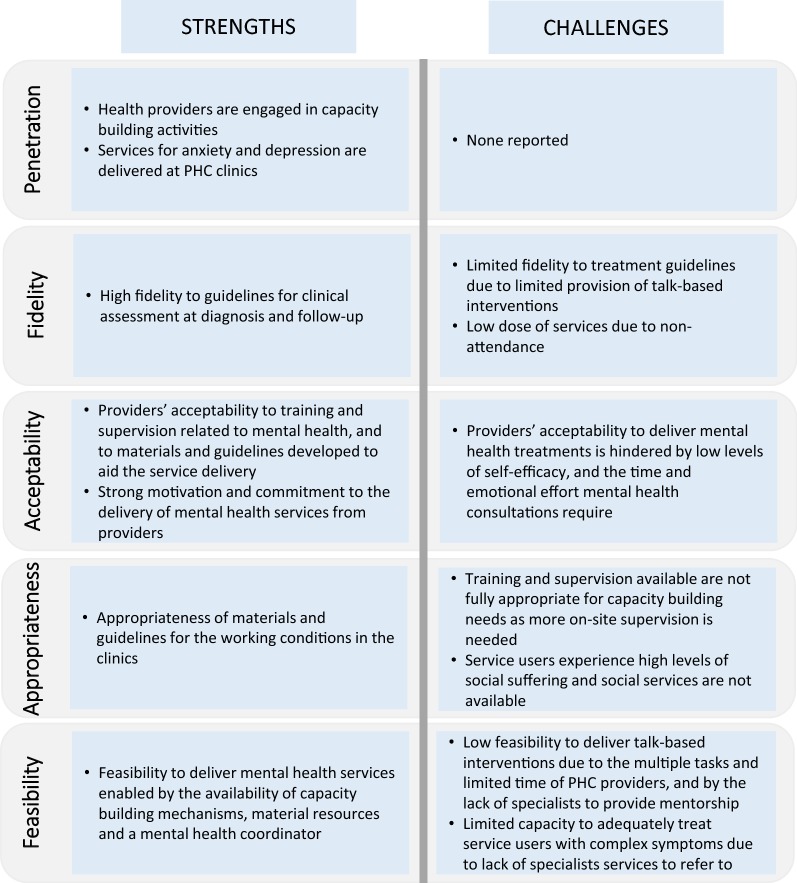
Summary of findings according to implementation outcomes

The CES programme was successful in terms of achieving the integration of mental health services into 10 PHC clinics located in a rural area of Chiapas, Mexico. A summary of programme strengths and remaining challenges can be found in Fig. 4. The penetration of programme activities was evidenced by the presence of capacity building activities and the routine delivery of mental health services, including identification of service users with mood or anxiety disorders, diagnosis, and treatment delivery. A key driver for penetration was the presence of a programme leader and a team that promoted the delivery of mental health services and provided continuous support to do this. The programme was also largely acceptable to providers, as evidenced by providers’ engagement and commitment to programme activities. Programme fidelity was not fully achieved given the low rates of attendance, and limited adherence to treatment guidelines. Low attendance can be attributed to challenges travelling to the clinics due to lengthy and costly journeys, as well as a lack of interventions that tackle the service users’ social needs (e.g. poverty and intimate partner violence). Adherence to treatment guidelines by MDs was limited, as talk-based interventions were not provided in the majority of consultations. Key challenges included the limited availability of training and on-site supervision by specialists, as well as limited time due to the numerous tasks that providers are responsible for, and the large patient loads at the clinics. Despite feasibility challenges, the essential support and resources provided by CES, including mentoring, guidelines, printed materials, and medications made the programme implementation possible.

A previous study in Mexico highlighted the difficulties of delivering mental health services in PHC due to an overwhelming lack of resources (i.e. budget constraints, lack of medications and sufficient human resources) at this level of care [21]. Our findings indicate that the presence of strategies that strengthen the service delivery platform (i.e. adequate financing, the inclusion of ongoing capacity building mechanisms, information systems to monitor progress and ensuring medication supply) are essential to implement mental health programmes in PHC [22, 23]. Similar to previous literature, we also found that important challenges that need to be addressed are frequent turnover of health personnel [22], the skills and time requirements to deliver talk-based interventions [24], and the need of specialists to support PHC providers and ensure that services delivered are of quality [25]. To overcome some of these challenges, besides adequate resourcing, committed leadership and support teams are also key to promote implementation and provide continuous assistance in this process [26, 27].

The essential need for capacity building mechanisms that include adequate and ongoing support and supervision, has been highlighted as relevant by other programmes integrating mental health services at PHC [28]. Previous research indicates that rather than single trainings, apprenticeship models are required for effective implementation [29]. According to our findings and previous research, capacity building can improve self-efficacy [24], and in this programme supervision was essential to help providers manage the stress caused by working in underserved areas where high levels of social suffering are witnessed. It is possible that the high levels of acceptability reported by health providers can also be attributed to the presence of capacity building mechanisms, which are dissimilar to what has been observed in other Mexican settings where levels of stigma from health providers’ are high [21]. Collaborative care models have been promoted as a solution to make more efficient use of resources and redistribute workloads [30]. Other programmes have pointed out that appropriate implementation of this model requires the recruitment of new cadres, e.g. CHWs, to deliver non-pharmacological treatments and manage the chronic care needed by mental health service users [31, 32]. Previous studies have also shown that the use of CHWs for the delivery of talk-based interventions is satisfactory and acceptable to service users [24], which can increase adherence [31]. Our findings also indicate that recruiting CHWs might be necessary to decrease the workload of PHC providers and deliver talk-based interventions more effectively (i.e. with higher frequency and improved adherence). Moreover, increasing the availability of psychiatrists and psychologists to support capacity building, and strengthening specialist services to refer those service users with complex needs are also necessary steps for successful implementation [33].

Finally, the role of intersectoral collaboration in tackling the social determinants of health has been previously emphasised [34], and it is especially important in settings where the risk of poor mental health is greater due to high levels of poverty and other social risk factors [35]. To appropriately tackle the social needs of service users we will need to develop targeted interventions that address gender-based violence, income and food insecurity, and other structural issues in these and other similar settings [36]. In this sense, there is an important role for the inclusion of links to social work interventions in the planning of PHC based programmes [37]. Furthermore, increasing community based services through CHWs and community participation is essential to accomplish better access to interventions that tackle both health and social needs [38, 39]. The outreach nature of community-based services delivered by CHWs can help overcome logistical challenges (e.g. travelling times, costs, and waiting times) that hinder attendance [40]. This is especially relevant in remote and rural settings with high-levels of poverty where even PHC clinics can be too hard to reach [41].

The current study has several strengths. We used a comprehensive methodology that both described the implementation of the mental health programme, and described how this was achieved [15], and selected implementation outcomes based on relevant frameworks [16, 19]. The selection criteria for both samples aimed to be as unrestrictive as possible to improve representativeness. The data collection was performed by researchers who spent at least a year in the field, which increased familiarity with the context and buy-in from the programme staff. Qualitative data was checked for quality, and translations of quotes were done by multiple researchers. We also ensured high quality of quantitative data by using several techniques, including cross checking between the HIS and other data collection tools, and double-coding of fidelity scores.

In terms of limitations, for the qualitative study we used a convenience sample due to time constraints, however we included 56% of the programme personnel. Administrative staff perform important activities for the programme, but none of them were included for practical reasons. Service user perspectives were also not included in the current analysis, but are presented elsewhere. For the quantitative component although there were very few instances of missing data due to provider error, there was a lack of standardized guidelines for recording data, which meant the quality of clinical notes was variable. Additionally, the clinical notes could not be interpreted as a perfectly faithful representation of all events that occurred during a consultation due to variability regarding what talk-based interventions entail. Finally, the generalizability of our findings might be limited given that providers’ of this programme report allocating between 15 and 20 min per consultation, which is significantly more that the average of 5 min found by previous research [42].

## Conclusions

The current study aimed to contribute to the scarce evidence base on implementation of mental health programmes integrated in PHC platforms in low resource settings, which is needed given the difficulties in translating policy into practice. This study demonstrates that it is possible to deliver certain mental health services at PHC platforms by non-specialists when adequate resources, support and supervision structures are in place, even in low-resource, rural, and remote settings. MDs identified service users with mental health conditions successfully and performed appropriate clinical assessments. However, talk-based interventions, an important element of programme guidelines, were rarely delivered. Fidelity to guidelines is constrained by the lack of mental health training MDs receive related to mental health in their professional education and the limited availability of mental health specialists to provide mentorship. The majority of service users did not attend more than one follow-up consultation. Distance and lack of social support services need to be tackled to increase the appropriateness of services for service user needs. Integration of mental health care services in PHC in Mexico will require improved financing and resource management of PHC and specialist services, ongoing capacity building, the development of effective referral systems, further development of community-based services, and to link PHC with locally-relevant social interventions.

## Supplementary information

**Additional file 1: Appendix S1.** Coding system to assess programme fidelity.

**Additional file 2: Appendix S2.** Interview guide.

## Data Availability

The data that support the findings of this study are available from Compañeros En Salud but restrictions apply to the availability of these data, which were used under license for the current study, and so are not publicly available. Data are however available from the authors upon reasonable request and with permission of Compañeros En Salud.

## Abbreviations

CES: Compañeros En Salud
GAD-7: 7-item Generalized anxiety disorder scale
HIS: Health information system
LMICs: Low- and middle-income countries
MD: Medical doctor
MRC: Medical Research Council
PHC: Primary Health Care
PHQ-9: 9-item Patient health questionnaire
WHO: World Health Organization
