# Supplementary material for: Implementation process and outcomes of a mental health programme integrated in primary care clinics in rural Mexico: a mixed-methods study
Source: Int J Ment Health Syst. 2020 Mar 16;14:21. doi: 10.1186/s13033-020-00346-x (PMC7074983; doi:10.1186/s13033-020-00346-x)
Supplement: Supplementary file 1 — Additional file 1: Appendix S1. Coding system to assess programme fidelity. [file 13033_2020_346_MOESM1_ESM.docx]

**Appendix S1. Coding system to assess programme fidelity**

| Indicator | Description | |
| --- | --- | --- |
| Fidelity to diagnostic guidelines | 1: Record of key symptoms and/or relevant life events and scale score (i.e. PHQ-9^21^ or GAD-7^22^)  2: Record of key symptoms and/or relevant life events, but scale score (i.e. PHQ-9^21^ or GAD-7^22^) not available  3: Scale score (i.e. PHQ-9^21^ or GAD-7^22^) available, but record of key symptoms and/or relevant life events missing | |
| Fidelity to treatment allocation guidelines | 1: Pharmacological treatment allocated only when scale score (i.e. PHQ-9^21^ or GAD-7^22^) is 15 or above unless service user rejects medication or MD decides to reassess need in a second appointment; if 14 or less, medication not prescribed until reassessment at second appointment; talk-based intervention provided  2: Medication provided following algorithm, but talk-based intervention not provided  3: Talk-based intervention provided, but medication not prescribed following above algorithm  4: Neither talk-based intervention provided nor medication provided according to algorithm | |
| Fidelity to clinical assessment guidelines at follow-up | (1) Scale score | 1: Scale score (i.e. PHQ-9^21^ or GAD-7^22^) collected during follow-up consultations with MDs |
|  | (2) Key symptom exploration | 1: Any notes about relevant symptoms picked up during assessment or the emotional status of the patient during the last month |
|  | (3) Life event exploration | 1: Any notes about any relevant positive or negative events during the last month |
| Fidelity to treatment allocation guidelines at follow-up | (1) Talk-based intervention | 1: Any notes about talk-based intervention provided to service user during follow-up consultation, e.g. psychoeducation, talk-based interventions or other advice |
