# Supplementary material for: Implementation process and outcomes of a mental health programme integrated in primary care clinics in rural Mexico: a mixed-methods study
Source: Int J Ment Health Syst. 2020 Mar 16;14:21. doi: 10.1186/s13033-020-00346-x (PMC7074983; doi:10.1186/s13033-020-00346-x)
Supplement: Supplementary file 2 — Additional file 2: Appendix S2. Interview guide. [file 13033_2020_346_MOESM2_ESM.docx]

**Appendix S2. Guide for semi-structured interviews with health workers**

Participants provided written informed consent prior to the start of data collection. Informed consent and data collection were undertaken in Spanish.

**Introduction:** *Thank you for agreeing to participate in this interview. Before we start, I would like to remind you that your participation in this interview is voluntary and you may stop participating whenever you wish to do so. This discussion is private and confidential; therefore, I will not share or discuss any of what is talked about here with anyone outside my research team. That said also be reminded that all information shared will be anonymised. Also, please note that this discussion will be recorded to facilitate its analysis.*

*This interview is about the mental health programme that is being implemented in the clinic. I would like to learn about your experience with this program. For example, your opinion of the training, the guidelines, your thoughts on your own skills and knowledge to implement it, and how much support and resources you feel you can access to provide mental health services. Your honesty and cooperation will be really helpful to improve this programme.*

| **Topic** | **Questions** |
| --- | --- |
| **Initial exploration**  **Purpose:** corroborate the participant is familiar with the programme | Did you receive training to provide mental health services? How long ago did you receive this training?  What was the content of the training?  What are the services you are supposed to provide/are providing?  To what extent was the training useful to provide mental health services? |
| **Topic relevance**  **Purpose:** Identify what determinants are more relevant to the participant | In relation to this programme, please tell me which from the following areas are the most relevant for the implementation of this programme?   - Guideline - Skills and knowledge to provide services - Capacity to discuss cases with others/refer patients to other services - Resources (time, medications, or others) - Capacity to implement the programme in the organization - Leadership   Which areas do you think are the most problematic/difficult?  Which do you think facilitate the implementation of the programme? |
| **Guideline/intervention acceptability, appropriateness and feasibility** | To what extent are you able to access the guideline when you need it?  What do you think of the guideline clarity? (i.e. is it understandable?)  What do you think of its suitability for the patients you work with?  What do you think of the content/usefulness of its recommendations?  What do you think of its appropriateness considering your available resources and training?  Is it compatible to the way you currently work or do you think its use/content is disruptive for your work flow?  What do you think of the amount of effort it requires?  To what extent can you observe the effects of the guideline in your work? |
| **Individual health professional capacity and willingness to deliver mental health services** | How familiar are you with the guideline content?  To what extent do you feel you have the skills to prescribe medication? Provide psychoeducation? Refer patients?  What are your feelings about providing treatment for patients with mental illness?  How confident do you feel to provide these treatments?  How do you feel about providing treatments for patients with mental health problems? |
| **Inner setting - readiness within the organization to implement the programme** | To what extent do you feel the programme is a priority in the organization?  What do you think about the leadership of the programme?  How able are you to express your opinion or concerns in the organization?  To what extent do you feel able to request more support?  What do you think are the necessary resources to implement the programme?  To what extent do you feel these are available?  To what extent do you consider the information system available is useful to implement the programme? (i.e. can you record and follow-up patients?)  To what extent do you feel you have sufficient support/assistance? |
| **Outer setting - links to other services and target population characteristics** | What are there referral systems in place?  What has been your experience using these systems?  How do the characteristics of the service users have an impact on the programme implementation? |
